# Retraction: Recombination spot identification Based on gapped k-mers

**DOI:** 10.1038/srep46940

**Published:** 2018-03-20

**Authors:** Rong Wang, Yong Xu, Bin Liu

Scientific Reports 6: Article number: 2393410.1038/srep23934; published online: 03 31 2016; updated: 12 07 2016; updated: 11 10 2017; updated: 03 20 2018

This Article reports an application of methodology originally reported in Reference 33 to recombination spot identification. Reference 33 (Ghandi, M. *et al. PLoS Comp. Biol.* [2014]) of this Article introduced a feature set called gapped k-mer for regulatory sequence prediction; this Article applied these gapped k-mer features to recombination spot identification, and a computational predictor was constructed for recombination spot identification.

The original and corrected versions of the Article include ambiguous sentences and textual overlap without adequate attribution, which failed to give due credit to the authors of Reference 33. The original and corrected versions of the Article are therefore being retracted by the Editors. The authors do not agree with the retraction.

